# Interferon (IFN)-gamma (γ) inducible protein 10 (IP-10) in the diagnosis of latent and active tuberculosis in Bacille Calmette Guerin (BCG)-vaccinated pediatric population

**DOI:** 10.1371/journal.pone.0314400

**Published:** 2025-01-21

**Authors:** Magdalena Druszczynska, Michał Seweryn, Sebastian Wawrocki, Anna Pankowska, Anastasiia Kulbachko, Magdalena Jurczak, Magdalena Kowalewska-Pietrzak

**Affiliations:** 1 Department of Immunology and Infectious Biology, Faculty of Biology and Environmental Protection, Institute of Microbiology, Biotechnology and Immunology, University of Lodz, Lodz, Poland; 2 Department of Molecular Biophysics, Faculty of Biology and Environmental Protection, Biobank Lab, University of Lodz, Lodz, Poland; 3 Regional Specialized Hospital of Tuberculosis, Lung Diseases, and Rehabilitation in Lodz, Lodz, Poland; 4 Lodz Institutes of the Polish Academy of Sciences, The Bio-Med-Chem Doctoral School, University of Lodz, Lodz, Poland; Stellenbosch University, SOUTH AFRICA

## Abstract

**Background:**

Accurate diagnosis of tuberculosis (TB) in children continues to be challenging, primarily due to the low bacterial load characteristic of the disease and the obstacles in collecting sputum samples. Furthermore, detecting cases of latent *Mycobacterium tuberculosis* (*M*.*tb*) infection (LTBI) that have a high risk of progressing to active TB disease remains a significant diagnostic hurdle.

**Objective:**

The study explored the utility of interferon-gamma (IFN-γ) inducible protein 10 (IP-10) for diagnosing latent and active *M*.*tb* infections among children vaccinated with the Bacille Calmette-Guerin (BCG) vaccine. The research specifically assessed IP-10 levels in serum, urine, and QuantiFERON-TB Gold Plus (QFT) cultures stimulated with *M*.*tb* antigens to determine if IP-10 could be a useful diagnostic marker for pediatric tuberculosis, either alongside or as an alternative to IFN-γ.

**Results:**

Both urine and QFT cultures stimulated with *M*.*tb* antigens showed significantly higher IP-10 levels in individuals with active TB or latent TB infection (LTBI) when compared to those uninfected by *M*.*tb* but with nonmycobacterial pneumonia (NMP) and healthy controls (HC). Similarly, IFN-γ levels in *M*.*tb*-stimulated QFT cultures were significantly higher in the TB and LTBI groups compared to the NMP and HC groups. Notably, the study found a significant difference in IFN-γ levels between the TB and LTBI groups in the QFT cultures, a distinction not observed for IP-10 concentrations. Serum levels of IP-10 and IFN-γ did not significantly vary across the study cohorts.

**Conclusions:**

IP-10 might be a viable alternative biomarker to IFN-γ for identifying *M*.*tb* infection in BCG-vaccinated children, although it cannot distinguish between latent and active TB cases. This highlights the potential of IP-10 in improving TB diagnosis among children, addressing the challenges posed by the paucibacillary nature of pediatric TB, but also underscores the need for further research to refine diagnostic approaches for distinguishing between latent and active TB infections.

## Introduction

Tuberculosis (TB), despite being preventable and treatable, remains a significant infectious threat to children worldwide. Annually, around 1 million children are affected by TB, representing about 11% of all TB cases, according to the World Health Organization’s 2023 report [1]. The WHO’s data also reveal that in 2021, TB claimed the lives of approximately 214,000 children under 15, highlighting the severity of this disease among young populations. Particularly vulnerable are infants and toddlers (aged 0–2 years), who face the highest mortality rates from TB. Children up to 5 years old infected with TB have a 19% risk of developing the active disease, a stark contrast to the 8–12% risk among those aged 5–19 years [2].

In Poland, the situation mirrors the global concern and offers a glimpse into the country-specific impact of TB. In 2021, the TB incidence rate was 9.7 per 100,000 people, marking a 10.2% increase from 2020, which had a rate of 8.8. Out of 3,704 TB cases registered that year, 37 were in children up to 14 years of age, accounting for 1.0% of the total cases [3]. Adolescents aged 15 to 19 saw 51 cases, resulting in an incidence rate of 2.8 per 100,000. These figures underscore the ongoing challenge TB poses, emphasizing the need for continued vigilance and intervention to protect vulnerable populations, especially children.

The diagnosis of TB in children poses unique challenges, as the presentation of the disease can often mimic other common pediatric infections, displaying a wide array of nonspecific signs and symptoms [4]. The absence of a universally accepted "gold standard" for diagnosis means clinicians frequently rely on a combination of factors, including contact history, clinical presentation, responses to the tuberculin skin test (TST), and chest X-ray findings [5]. The difficulties in diagnosing TB in children are compounded by the disease’s paucibacillary nature and the challenges in obtaining suitable diagnostic samples [6]. Despite advances in molecular diagnostics, these limitations persist, underlining the need for improved diagnostic strategies.

Immunodiagnostic tests, such as interferon-gamma (IFN-γ) release assays (IGRAs), represent a step forward, offering better specificity than the TST for detecting *M*.*tb* infection. However, these tests, including QuantiFERON TB Gold In Tube and T-SPOT.TB, do not differentiate between latent *M*.*tb* infection and active TB disease [7]. The accurate identification of active versus latent TB is crucial not only for administering the appropriate treatment to those with active disease but also for providing preventive care to individuals with latent infections to avert disease activation. This underscores the importance of developing new markers for protective immunity or indicators of disease progression as integral components of global TB control efforts. The pursuit of these advancements remains a priority in the ongoing battle against TB, emphasizing the need for continued research and innovation in the field.

In the quest for more effective TB diagnostics, recent research has spotlighted CXCL10 (CXC motif ligand 10), also known as IFN-γ-inducible protein 10 (IP-10), as a potentially valuable biomarker. CXCL10 is a pro-inflammatory chemokine released by various cell types upon IFN-γ stimulation, facilitating the recruitment of monocytes and activated Th1 lymphocytes to inflammation sites through CXCR3 interaction. Additionally, IP-10 enhances Th1 immune responses by promoting IFN-γ expression and plays a role in delayed-type hypersensitivity [8, 9]. Research into IP-10’s diagnostic utility for TB, encompassing both adults and children, has produced mixed outcomes. Elevated levels of IP-10 have been detected in the serum, plasma, and urine of adults with active TB [10–13]. Conversely, the sensitivity of IP-10 for identifying *M*.*tb* infection among children with active TB has been variably reported, with some studies noting limited sensitivity in both low- and high-TB endemic regions [7, 14, 15]. Moreover, IP-10 has been suggested as a potential indicator for monitoring the efficacy of anti-TB treatment. Sequential measurements have shown a decrease in IP-10 levels following two months of therapy, indicating its possible role in assessing treatment response [16].

This backdrop sets the stage for the current investigation, aimed at exploring the diagnostic capabilities of IP-10 for latent and active *M*.*tb* infections among Polish children and adolescents immunized with the Bacille Calmette-Guerin (BCG) vaccine. By comparing IP-10 concentrations in serum, urine, and IGRA supernatants, this study seeks to determine whether IP-10 can serve as a diagnostic marker for pediatric TB, either as a standalone measure or in conjunction with IFN-γ. This exploration into IP-10’s potential underscores the ongoing efforts to refine diagnostic strategies for TB, particularly in pediatric populations where accurate diagnosis remains a complex challenge.

## Materials and methods

### Study population

A total of 235 HIV-negative Polish children and adolescents, aged 1–17, vaccinated after birth with *Mycobacterium bovis* BCG Moreau according to the Polish national vaccination program, were included in the study. The study was conducted in patients hospitalized between January 2017 to December 2019 at the Regional Specialized Hospital of Tuberculosis, Lung Diseases and Rehabilitation in Lodz, Poland. The protocol for the cross-sectional study was approved by the Research Ethics Committee of the Medical University in Lodz (no. RNN/138/15/KE).

After obtaining written informed consent from the children’s parents or guardians, children underwent a medical interview, physical examination, and clinical and radiological evaluation including chest X-ray, tuberculin skin testing, and IGRA (interferon gamma release assay) testing with QuantiFERON-TB Gold Plus assay (QFT). In children with symptoms of lower respiratory tract infections the differential diagnosis was performed, including diagnostic tests for TB. For this purpose, gastric aspirates or bronchoaspirates collected from the children were examined using standard microbiological methods including Ziehl-Neelsen staining (smears), culturing on liquid (BACTEC MGIT 960 system) and solid (Löwenstein-Jensen) media, and genetic testing with the use of the GeneXpert MTB/RIF molecular system. Based on the complex analysis of the results of the clinical examination, the children were divided into groups: Group 1, including children with active TB (*M*.*tb* culture positive), Group 2, including children with LTBI (IGRA positive), Group 3, including children without TB and without LTBI (IGRA negative), suffering from acute nontuberculous pneumonia (NMP), Group 4, including healthy children with no signs or symptoms of any pulmonary diseases (IGRA negative). At the time of blood sampling, none of the participants had been treated with steroids or other immunosuppressive or antituberculous drugs. Demographic characteristics of the study cohort are shown in Table 1. The median age of the TB children was significantly higher than that of the LTBI or HC groups (p<0.05). There were no significant differences between the groups of the study regarding the sex.

**Table 1:** Demographic characteristics of the groups of the study.

Characteristic	Group 1	Group 2	Group 3	Group 4
	TB	LTBI	NMP	HC
Total, n	16	61	22	136
Age (years)				
median	15*	7*/**	15**	6*/**
IQR	14–17	4–17	7–17	3–17
Age range, n (%)				
<2 years	1 (6%)	6 (10%)^#/##^	1 (4%)	33 (24%)^#/##^
3–5 years	0 (0%)	15 (25%)	1 (4%)	28 (20%)
6–10 years	2 (12%)	21 (34%)	5 (23%)	44 (33%)
>10 years	13 (82%)	19 (31%)	15 (69%)	31 (23%)
Sex				
Girls, n (%)	9 (56%)	33 (54%)	14 (64%)	60 (44%)
Boys, n (%)	7 (44%)	28 (46%)	8 (36%)	76 (56%)

Abbreviations: IQR–interquartile range; n- number; HC–*M*.*tb*-uninfected healthy controls; LTBI–latently *M*.*tb* infected individuals; NMP- *M*.*tb* uninfected subjects with nonmycobacterial pneumonia; TB–tuberculosis patients.

There was a significant difference between the TB and LTBI or HC groups as well as between the NMP and LTBI or HC groups in terms of age (Kruskal-Wallis ANOVA, p<0.0001), however there was no differences between regarding the sex rate (χ2 test, p = 0.95) (Table 1). The percentage of the youngest participants in the study (< 2 years) was significantly higher in the HC group than in the LTBI (χ2 test, p = 0.05) or NMP (χ2 test, p = 0.02) groups. *p<0.0001 (Kruskal-Wallis ANOVA); **p<0.0001 (Kruskal-Wallis ANOVA); ^#^p = 0.05 (χ2 test); ^##^p = 0.02 (χ2 test).

### QuantiFERON-TB Gold Plus assay (QFT) and measurement of IFN-γ in QFT cultures

Prior to the tuberculin skin test, blood was drawn for the QuantiFERON-TB (QFT) Gold Plus assay (Qiagen, Hilden, Germany). According to the manufacturer’s instructions, four distinct collection tubes, including a Nil (negative control) tube, a TB1 Antigen tube, a TB2 Antigen tube, and a Mitogen (positive control) tube, were filled with one ml of blood. The tubes were then centrifuged at 2500 RCF for 15 min after incubation at 37°C for 16–20 hours. The level of IFN-γ in the resulting plasma was determined by ELISA. Briefly, in the first step of the assay, 50 μl of a diluted solution of mouse anti-human IFN-γ antibody conjugate with HRP was applied to the wells, and then 50 μl of each dilution of the IFN-γ standard or tested samples (Nil, TB1, TB2 and Mitogen) from each patient were added to the wells in duplicate. After application, the plate was covered with a lid, shaken on a microplate shaker for 1 min and then incubated at room temperature and in the dark for 2 h. After washing the wells 6 times with wash buffer, 100 μl of Enzyme Substrate Solution (Enzyme Substrate Solution) was added to each well, and the plates were incubated for 30 min at room temperature in the dark. After this time, 50 μl of the Enzyme Stopping Solution was added to each well, and then the optical density (OD) of each sample was measured using a multipurpose counter Victor 2 (Wallac Oy, Turku, Finland) equipped with a 450 nm filter. Using the QFT-Plus Analysis Software (ver. 2.71.2), the value of the IFN-γ concentration in each test sample was calculated based on the standard curve created for each assay. The test was considered positive when the IFN-γ value in both TB1 Antigen and TB2 Antigen minus Nil was greater or equal to 0.35 IU/ml. The concentration of IFN-γ is reported in pg/ml according to the international reference standard for human IFN-γ as presented by Desem and Jones [17]. According to this standard, 1 IU/ml corresponds to 40 pg/ml of human IFN-γ /ml.

### Measurement of IP-10 in serum, urine and whole blood QFT cultures

The IP-10 concentrations in the samples of sera, OFT supernatants and urine were measured using a Human CXCL10/IP-10 DuoSet® ELISA (R&D Systems, Minneapolis, USA) according to the manufacturer’s instructions. Samples were diluted 1:2 in assay diluent (10% bovine serum albumin (BSA) in phosphate buffered saline (PBS)) in order to optimise the expected IP-10 concentrations to the range of the standard curve and tested in duplicates. In brief, 50 μl of a diluted Human IP-10 Capture Antibody (2 μg/ml) was added to each well and incubated overnight at room temperature. After washings, 150 μl of assay diluent was added to block the plates and then incubated for 1 hour at room temperature. The plates were washed and then 50 μl of the recombinant Human IP-10 standard in the concentration range of 2,000 pg/ml-31.3 pg/ml or diluted samples was added to the wells and incubated for 2 hours at room temperature. After washing, 50 μl of Human IP-10 Detection Antibody (12.5 ng/ml) was added and incubated for 2 hours at room temperature. In the next step, 50 μl/well of streptavidin coupled with horseradish peroxidase (Streptavidin-HRP), diluted 1:40 in assay diluent was added and incubated for 20 min at room temperature. Finally, 50 μl of substrate solution (tetramethylbenzidine (TMB) + hydrogen peroxide (H_2_O_2_), 1:1) was added to the wells and incubated 20 minutes at room temperature in the dark. The enzymatic reaction was stopped by adding 25 μl of 1M H_2_SO_4_ solution to the wells. The well optical densities (OD) were read at 450 nm within 30 minutes using an ELISA plate reader (Wallac Oy, Turku, Finland).

### Measurement of IFN-γ in serum samples

A Bio-Plex Pro™ Human Th17 multiplex assay purchased from Bio-Rad (Hercules, CA, USA) was used to measure IFN-γ in serum samples. Briefly, pre-wet wells received 50 μl of beads, which were then washed twice. The plate was incubated for one hour after adding 50 μl of standard, internal control or sample. After washing, 25 μl of detection antibody solution was added to each well. In the next step, 50 μl of streptavidin-PE was added, and after a 10-minute incubation and washing of the plate, the beads were resuspended in 125 μl of assay buffer. A Bio-Plex MAGPIX^TM^ Multiplex Reader (Bio-Rad) was used to read the assays, and Bio-Plex Manager 5.0 software was used for data collection. A five-parameter logistic regression (5PL) formula was used to generate a standard curve to assess IFN-γ concentrations in the samples.

### Tuberculin skin testing

The dorsal side of the lower arm was given an intracutaneous injection of 2 tuberculin units of purified protein derivative (PPD) RT 23 (Statens Serum Institut, Copenhagen, Denmark). The diameter of the skin induration was measured 72 hours later, using a cut-off of 10 mm for positive response.

### Statistical analysis

Statistical analysis of the results was performed using GraphPad Prism 8.2.1. software (GraphPad Software, San Diego, CA). The non-parametric chi-square test or Fisher’s exact test was used to assess the statistical significance of the differences in the categorical variables. Non-parametric Kruskal-Wallis Anova with Dunn’s post-test comparison was used to compare IP-10 and IFN-γ results in the HC, MP, LTBI and TB groups. The Mann-Whitney U-test was used to compare the data between the cohorts. A p-value < 0.05 was considered significant. Receiver operating characteristic (ROC) analysis was performed to define the diagnostic potential of proteins by deriving the area under curve (AUC) sensitivity and specificity at their 95% confidence intervals and best cut offs to differentiate the study groups. For the comparison of multivariate classification models we utilize the gradient boosting technique (as implemented in the XGboost package in R). The multiclass classification model is based on the softmax function and cross entropy loss function. In the binary classification models we were using logistic transformation and logloss function. All class labels were assigned according to the max probability rule. The confusion matrix and performance metrics were estimated as implemented in the package caret. Feature importance was evaluated via the xgb.importance function.

## Results

### IP-10 and IFN-γ concentrations in serum, M.tb antigen-stimulated whole blood QFT cultures and urine

**Fig 1:**
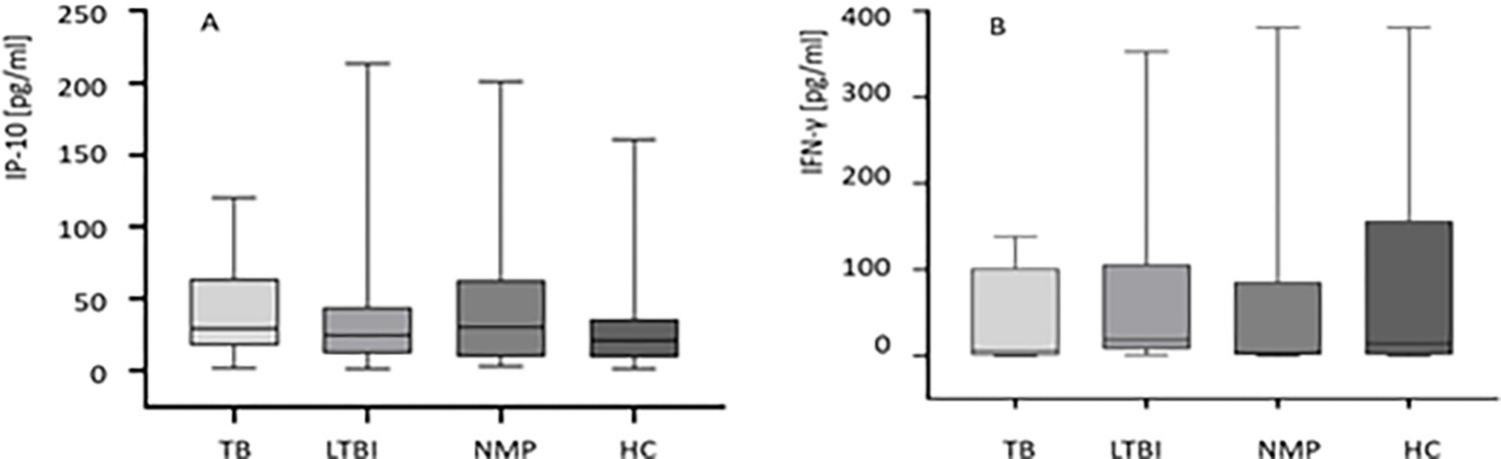
Serum IP-10 (A) and IFN-γ (B) levels in the groups of the study. Abbreviations: HC–healthy controls, IFN-γ–interferon-gamma, IP-10 –IFN-γ inducible protein 10, LTBI–latent *M*.*tb* infection, NMP–nonmycobacterial lung disease, TB- tuberculosis. The boxes show the median and interquartile range, and the whiskers show minimum and maximum values. Differences IFN-γ and IP-10 concentrations between the groups were compared using the non-parametric Kruskal-Wallis ANOVA with Dunn’s post-test. A p value was considered significant if < 0.05.

We compared the median serum concentrations of IP-10 and IFN-γ in children from the TB, LTBI, NMP and HC groups. As shown in Fig 1, serum levels of both proteins were not significantly different between the study groups. The median levels of IP-10 in the sera were 29.3 (IQR 17.6–69.8) in the TB group, 24.5 (IQR 21.3–31.3) in the LTBI group, 30.0 (IQR 10.1–59.1) in the NMP group, and 20.8 (IQR 18.5–25.1) in the HC group (Fig 1A). The median serum IFN-γ concentrations in each group was similar, at 2.65 (IQR 0.00–42.2), 3.73 (IQR 0.00–14.7), 3.72 (IQR 0.93–82.5), and 1.81 (1.1–15.5) in the TB, LTBI, NMP and HC groups, respectively (Fig 1B).

**Fig 2:**
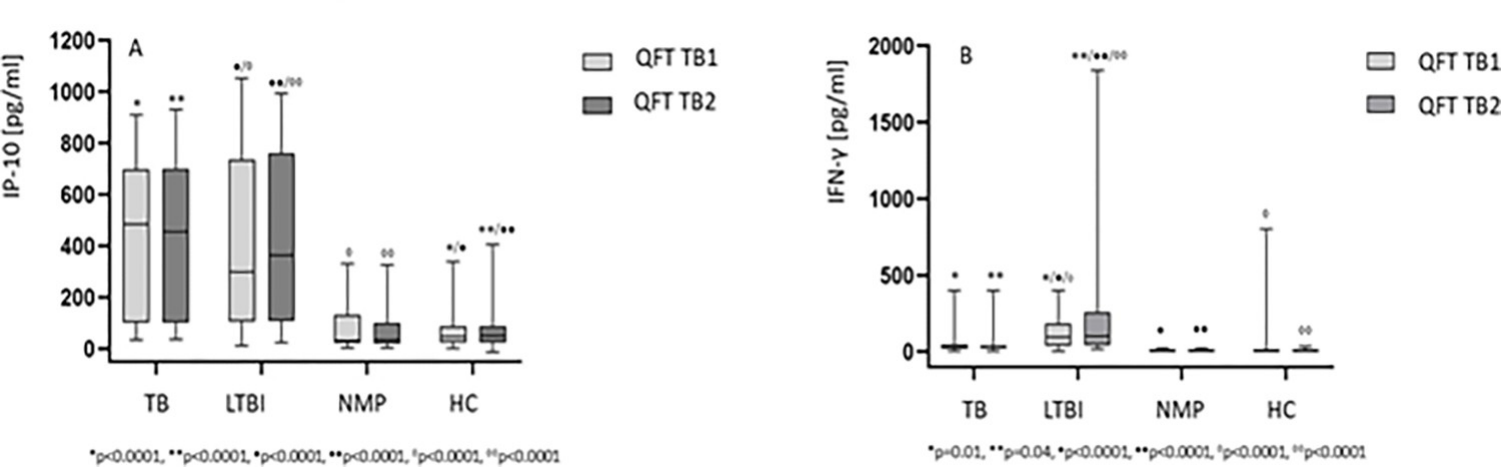
IP-10 and IFN-γ concentrations in *M*.*tb* antigen-stimulated whole blood QFT cultures. The boxes show the median and interquartile range, and the whiskers show the minimum and maximum values of IP-10 (A) or IFN-γ (B) in QFT TB1 (light grey) and QFT TB2 (dark grey) cultures. Statistical analysis was performed using the non-parametric one-tailed ANOVA. Lines represent statistically significant differences between studied groups. A p value was considered significant if < 0.05. Abbreviations: IFN-γ–interferon-gamma, IP-10 –IFN-γ inducible protein 10, QFT TB1—QuantiFERON-TB Gold Plus TB1 culture, QFT TB2—QuantiFERON-TB Gold Plus TB2 culture. HC–healthy controls, LTBI–latent *M*.*tb* infection, NMP–nonmycobacterial lung disease, TB- tuberculosis.

Differences in the median levels of IP-10 and IFN-γ in *M*.*tb* antigen-stimulated whole blood QFT cultures between the four study groups were determined by applying one-tailed ANOVA. The results revealed statistically significant differences for IP-10 in both QFT TB1 (p<0.0001) and QFT TB2 (p<0.0001) cultures between the HC group and the active TB and LTBI groups (Fig 2A). The levels of IP-10 in patients with active TB (QFT TB1: Me = 485.2, IQR 93.1–713.1; QFT TB2: Me = 455.3, IQR 102.9–700.1) and in subjects with LTBI (QFT TB1: Me = 299.6, IQR 117.6–678.7; QFT TB2: Me = 364.1, IQR 110.6–629.9) were significantly increased (p<0.001) compared to NMP (QFT TB1: Me = 32.7, IQR 23.6–100.1; QFT TB2: Me = 34.2, IQR 22.0–88.1) and HC (QFT TB1: Me = 46.9, IQR 40.4–57.5; QFT TB2: Me = 50.6, IQR 39.3–58.3) groups. As for IFN-γ, statistically significant differences were observed in QFT TB1 and QFT TB2 cultures between the HC and the LTBI groups (p<0.0001) as well as the TB and the LTBI cohorts (QFT TB1 p = 0.01; QFT TB2 p = 0.04) (Fig 2B). The median concentrations of IFN-γ were 28.0 (IQR 16.8–49.8) and 30.8 (IQR 20.4–41.2) in the TB group, 94.4 (IQR 60.0–134.8) and 100.4 (IQR 80.4–156.8) in the LTBI group, 5.6 (IQR 4.0–9.6) and 4.8 (IQR 4.0–10.8) in the NMP group, 5.6 (IQR 5.2–6.0) and 5.6 (IQR 5.2–6.0) in the HC group, in QFT TB1 and QFT TB2 cultures, respectively.

**Fig 3:**
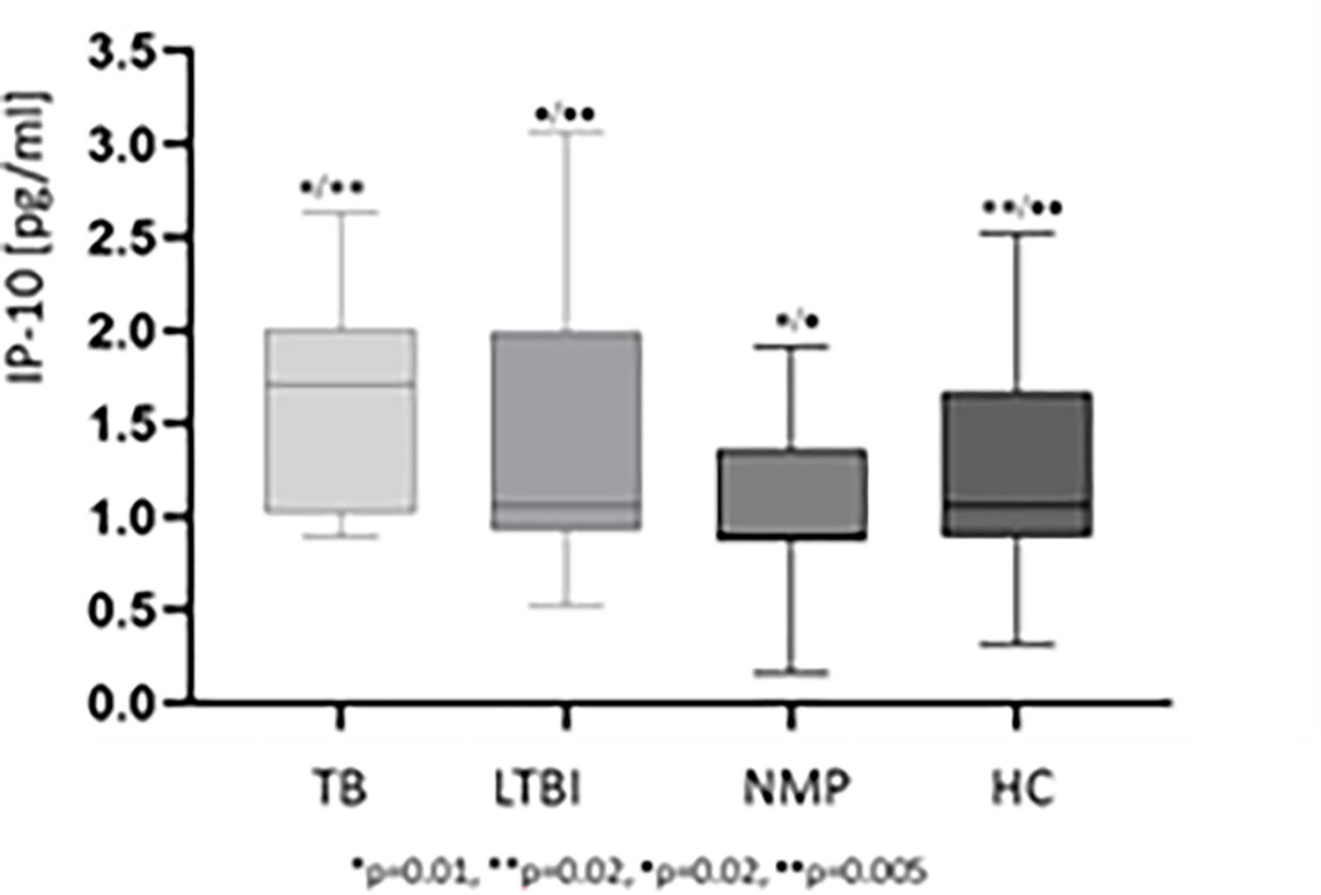
IP-10 concentrations in urine. The boxes show the median and interquartile range, and the whiskers show the minimum and maximum values of IP-10. Statistical analysis was performed using the non-parametric one-tailed ANOVA. A p value was considered significant if < 0.05. Abbreviations: HC–healthy controls, IP-10 –IFN-γ inducible protein 10, LTBI–latent *M*.*tb* infection, NMP–nonmycobacterial lung disease, TB- tuberculosis.

As shown in Fig 3, the levels of IP-10 in urine were significantly higher in the active TB (Me = 1.7, IQR 1.02–2.02) and LTBI (Me = 1.06, IQR 1.02–1.66) groups compared with the NMP (Me = 0.89, IQR 0.87–1.26) and HC (Me = 1.04, IQR 1.02–1.08) cohorts.

### IP-10 and IFN-γ responses among the different age groups

The study will analyse IP-10 and IFN-γ responses in children stratified into distinct age groups: ≤ 2 years, 3–5 years, 6–10 years, > 10 years, as proposed by Cuevas et al [18]. The stratification is crucial due to the differences and dynamics of the nature of TB disease across age [19]. Analysis IP-10 and IFN-γ levels in serum, urine, QFT TB1 and QFT TB2 cultures in the four age ranges of the children studied showed similar median concentration of the proteins studied in each age category (Table 2), and the Spearman’s rank correlation test confirmed a lack of association between the age and the concentrations of IP-10 and IFN-γ in all the samples studied (S1 Fig).

**Table 2:** IP-10 and IFN-γ levels among the different age groups.

Sample	Protein	Age range (Me, IQR)			
		< 2 years	3–5 years	6–10 years	>10 years
serum	IP-10	20.1 (12.6–30.3)	22.7 (16.3–29.3)	23.9 (19.5–30.8)	24.5 (17.6–31.3)
	IFN-γ	1.2 (0.6–11.4)	0.93 (0.66–16.9)	3.7 (0.98–42.2)	3.7 (1.02–14.8)
QFT TB1	IP-10	49.6 (37.2–80.2)	86.4 (44.2–107.6)	71.8 (46.5–92.7)	103.1 (55.9–113.2)
	IFN-γ	6.4 (5.2–9.6)	7.2 (4.8–23.2)	7.2 (5.6–9.2)	10.0 (7.2–18.8)
QFT TB2	IP-10	54.6 (35.5–82.9)	75.2 (47.2–104.0)	68.2 (49.7–92.1)	101.8 (50.6–114.1)
	IFN-γ	6.4 (5.2–8.0)	9.0 (5.2–25.2)	6.8 (5.6–10.4)	12.0 (6.8–20.8)
urine	IP-10	1.1 (0.98–1.19)	1.06 (1.03–1.5)	1.03 (1.01–1.09)	1.02 (0.93–1.13)

Abbreviations: IFN-γ–interferon-gamma, IP-10 –IFN-γ inducible protein 10, IQR—interquartile range, Me–median. Differences in IFN-γ and IP-10 concentrations between the groups were compared using the non-parametric one-tailed ANOVA. A p value was considered significant if < 0.05.

### IP-10 and IFN-γ responses in children with a positive (TST-positive) or negative (TST-negative) skin test reaction to tuberculin

**Fig 4:**
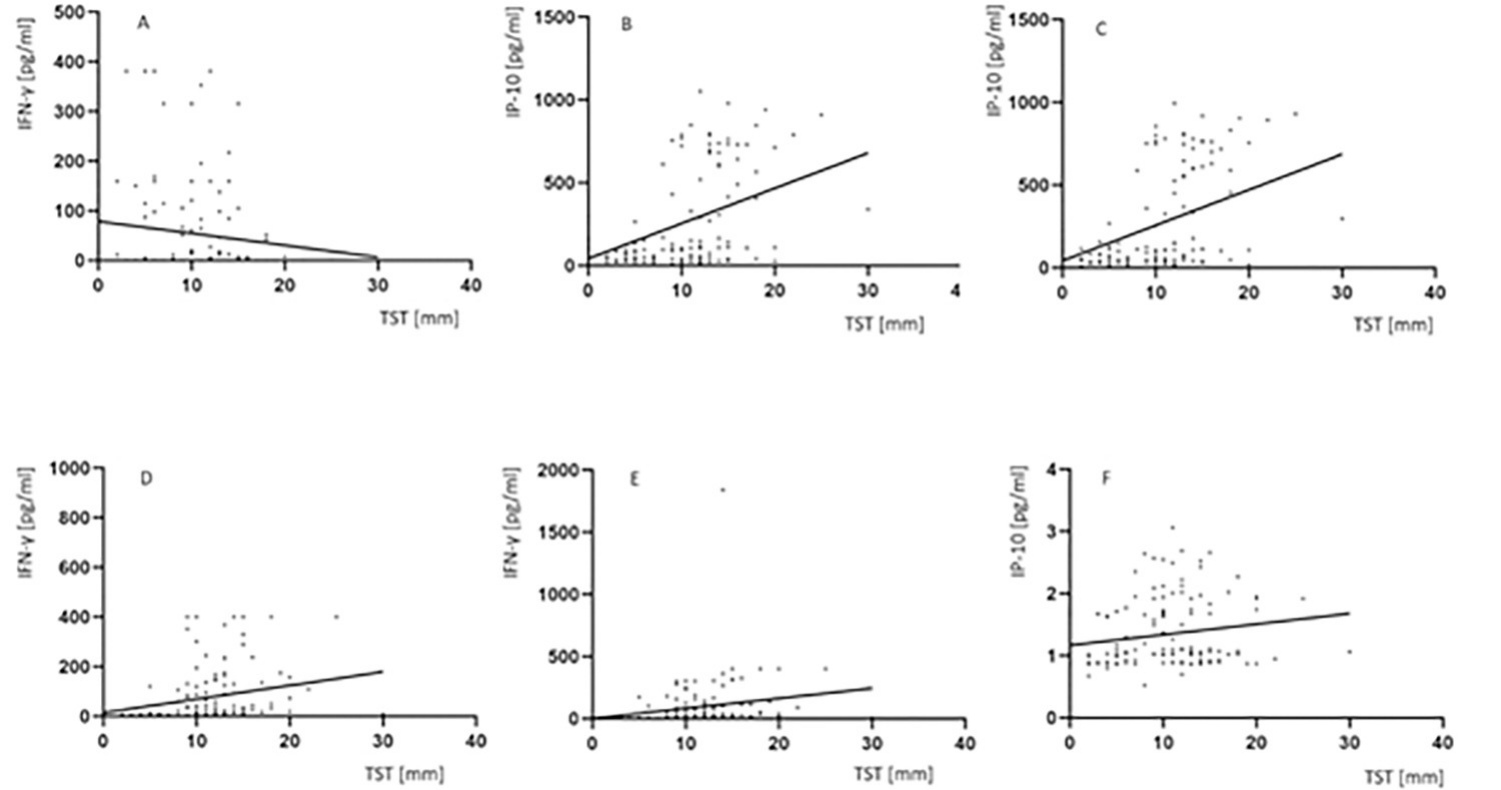
Correlation between the TST size of the levels of IP-10 or IFN-γ measured in serum, urine, QFT TB1 and QFT TB2 cultures. (A) Spearman rank correlation (r_s_) of TST size and serum IFN-γ levels (r = -0.2, p = 0.008), (B) Spearman rank correlation (r_s_) of TST size and QFT TB1 IP-10 levels (r_s_ = 0.44, p<0.0001), (C) Spearman rank correlation (r_s_) of TST size and QFT TB2 IP-10 levels (r_s_ = -0.44, p<0.0001), (D) Spearman rank correlation (r_s_) of TST size and QFT TB1 IFN-γ levels (r_s_ = 0.62, p<0.0001), (E) Spearman rank correlation (r_s_) of TST size and QFT TB2 IFN-γ levels (r_s_ = 0.63, p<0.0001), (F) Spearman rank correlation (r_s_) of TST size and urine IP-10 levels (r_s_ = 0.2, p = 0.002). Abbreviations: IFN-γ–interferon-gamma, IP-10 –IFN-γ inducible protein 10. Statistical analysis was performed using the Spearman’ rank correlation test and p value was considered significant if < 0.05.

As presented in Fig 4, we noted that there was a significant difference (p<0.0001) between the studied groups in the median TST size (TB: Me = 16.5, IQR 10.0–20.0; LTBI: Me = 12.0, IQR 11.0–13.0; NMP: Me = 0, IQR 0–5.0; HC: Me = 0, IQR 0–2.0) (S2 Fig). The median levels of IP-10 and IFN-γ in serum, urine, QFT TB1 and QFT TB2 cultures in children with positive or negative skin reaction to tuberculin is showed in Table 3. There were no differences in serum or urine protein concentrations in TST+ and TST- subjects from either group. However, in the group of children with active TB, the median levels of IP-10 and IFN-γ in QFT TB1 and QFT TB2 cultures were significantly higher in TST+ individuals compared to the TST- children (Table 3). The levels of proteins in QFT TB1 and QFT TB2 cultures from the TST-positive and TST-negative children from LTBI, NMP and HC groups were comparable. The Spearman’s rank correlation test confirmed the association between the magnitude of TST and the concentrations of IP-10 measured in QFT TB1 (r_s_ = 0.44, p<0.0001) and QFT TB2 (r_s_ = 0.44, p<0.0001) cultures and in urine (r_s_ = 0.2, p = 0.002) (Fig 4B, 4C and 4F). In addition, there was a correlation was found between the TST size and IFN-γ levels in serum (r_s_ = -0.2, p = 0.008), QFT TB1 (r_s_ = 0.62, p<0.0001) and QFT TB2 (r_s_ = 0.63, p<0.0001) cultures (Fig 4A, 4D and 4E).

**Table 3:** IP-10 and IFN-γ levels in the groups with a positive (TST+) or negative (TST-) skin test reaction to tuberculin.

Sample	IP-10		IFN-γ	
Group	(Me, IQR)		(Me, IQR)	
	TST-	TST+	TST-	TST+
serum				
TB	18.5 (13.5–69.8)	36.4 (17.6–73.7)	2.6 (1.0–132.7)	2.15 (0–42.2)
LTBI	23.0 (11.1–50.5)	25.8 (21.3–31.3)	3.1 (0.93–105.8)	1.91 (0–12.4)
NMP	34.4 (18.7–59.1)	25.9 (5.3–149.9)	3.7 (0.93–87.4)	14.2 (0.8–82.5)
HC	22.7 (19.0–27.9)	16.3 (8.8–22.7)	1.8 (1.2–15.5)	6.9 (0–159.6)
QFT TB1				
TB	63.3 (33.4–93.1)*	589.1 (106.6–713.1)*	9.6 (4.0–12.0)**	32.4 (19.6–72.8)**
LTBI	150.9 (87.7–612.0)	452.2 (113.2–731.1)	80.4 (32.8–131.2)	109.2 (58.8–151.6)
NMP	31.2 (15.4–74.1)	140.9 (26.5–329.6)	4.8 (4.0–6.0)	13.2 (7.2–25.2)
HC	51.3 (42.2–63.6)	40.0 (24.4–47.4)	5.2 (4.8–6.0)	6.8 (5.2–10.0)
QFT TB2				
TB	97.4 (36.8–134.8)***	574.3 (107.4–748.3)***	8.4 (3.2–14.0)****	35.2 (23.6–51.6)****
LTBI	123.2 (91.1–588.0)	526.0 (110.6–720.5)	93.6 (49.2–180.4)	100.4 (76.0–157.6)
NMP	30.0 (9.4–72.4)	111.5 (18.2–327.0)	4.4 (4.0–6.4)	18.0 (1.6–24.0)
HC	52.9 (36.7–60.3)	43.7 (26.4–62.8)	5.6 (5.2–6.0)	6.2 (4.8–9.6)
urine				
TB	1.7 (1.1–2.6)	1.7 (1.0–2.0)	-	-
LTBI	1.5 (1.0–2.1)	1.1 (1.0–1.7)	-	-
NMP	0.9 (0.9–1.6)	0.9 (0.9–1.3)	-	-
HC	1.0 (0.9–1.0)	1.6 (1.0–1.7)	-	-

*p = 0.04

**p = 0.003

***p = 0.04

****p = 0.0003

Abbreviations: IFN-γ–interferon-gamma, IP-10 –IFN-γ inducible protein 10, IQR—interquartile range, Me–median. Differences in IFN-γ and IP-10 concentrations between TST- and TST+ groups were compared using the non-parametric U Mann-Whitney test. A p value was considered significant if < 0.05.

### Relationship between IP-10 and IFN-γ responses in serum and QFT blood cultures

**Fig 5:**
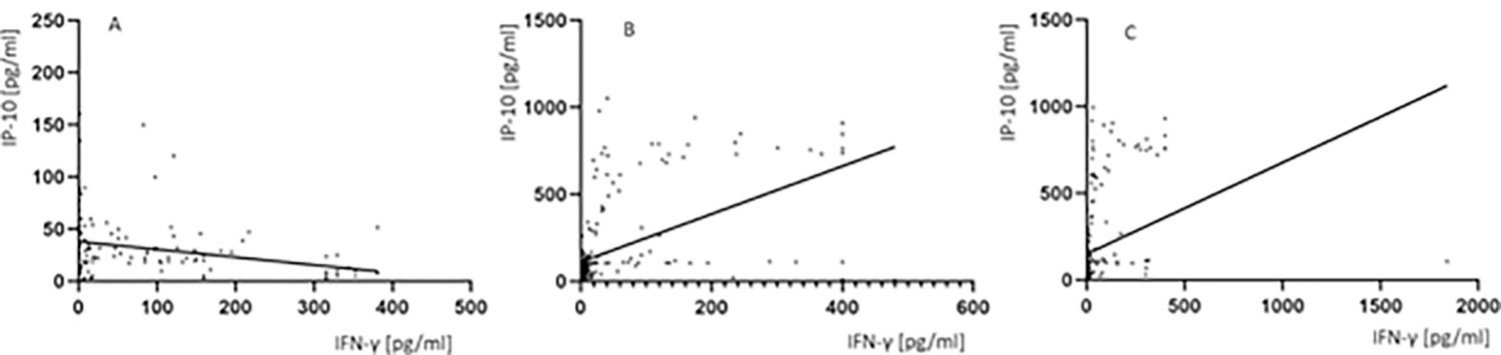
Correlation between the levels of IP-10 and IFN-γ measured in serum (A), QFT TB1 (B) and QFT TB2 (C) cultures. (A) Spearman rank correlation (r_s_) of serum IP-10 and IFN-γ levels (r = -0.24, p = 0.0005), (B) Spearman rank correlation (r_s_) of IP-10 and IFN-γ levels in QFT TB1 culture (r_s_ = 0.58, p<0.0001), (C) Spearman rank correlation (r_s_) of IP-10 and IFN-γ levels in QFT TB2 culture (r_s_ = 0.57, p<0.0001). Abbreviations: IFN-γ–interferon-gamma, IP-10 –IFN-γ inducible protein 10. Statistical analysis was performed using the Spearman’ rank correlation test and p value was considered significant if < 0.05.

Generally, there was a good correlation between IP-10 and IFN-γ levels measured in serum (r_s_ = -0.24, p = 0.0005), QFT TB1 (r_s_ = 0.58, p<0.0001) and QFT TB2 (r_s_ = 0.57, p<0.0001) cultures (Fig 5) and this pattern was similar among the groups of healthy controls, latently infected individuals, patients with active TB, and *M*.*tb* uninfected subjects with nonmycobacterial pneumonia.

### Diagnostic potential of IP-10 as a biomarker in the diagnosis of active and latent TB

For the evaluation of the potential of IP-10 measured either in serum or urine or *M*.*tb*-stimulated whole blood QFT cultures enabling discrimination between the study groups, receiver operating characteristic curves were plotted (S3–S6 Figs), and the area under the curve (AUC) was determined and the following comparisons: (1) HC vs. TB+LTBI+NMP, (2) TB vs. LTBI+NMP+HC, (3) TB+LTBI vs. HC+NMP, and (4) TB+NMP vs. HC+LTBI (Table 4). The highest AUC values in the TB vs. LTBI+NMP+HC comparison were observed for IP-10 measured in QFT TB1 (0.750) or QFT TB2 (0.756) cultures. IP-10 measured in *M*.*tb*-stimulated whole blood QFT cultures also showed the highest AUCs in the TB+LTBI vs. HC+NMP comparison, 0.882 and 0.868 in QFT TB1 and QFT TB2 cultures, respectively. AUC values for IP-10 measured in sera or urine were similar in each of the comparisons tested (Table 4).

**Table 4:** Results of the ROC analyses of IP-10 and IFN-γ measured in sera, urine and QFT cultures.

Sample	Protein	AUC			
		HC vs. TB+LTBI+NMP	TB vs. LTBI+NMP+HC	TB+LTBI vs. HC+NMP	TB+NMP vs. HC+LTBI
serum	IP-10	0.583	0.585	0.554	0.602
	IFN-γ	0.551	0.568	0.588	0.512
QFT TB1	IP-10	0.795	0.750	0.882	0.524
	IFN-γ	0.858	0.724	0.962	0.501
QFT TB2	IP-10	0.773	0.756	0.868	0.515
	IFN-γ	0.870	0.729	0.981	0.509
urine	IP-10	0.573	0.694	0.630	0.509

Abbreviations: AUC–area under curve; IFN-γ–interferon-gamma, IP-10 –IFN-γ inducible protein 10; HC–*M*.*tb*-uninfected healthy controls; LTBI–latently *M*.*tb* infected individuals; NMP–patients with nonmycobacterial pneumonia; TB–active tuberculosis patients.

To compare the diagnostic potential of IP-10 with IFN-γ, AUC values for IFN-γ assessed in serum or QFT whole blood cultures stimulated with *M*.*tb* antigens for comparisons: (1) HC vs. TB+LTBI+NMP, (2) TB vs. LTBI+NMP+HC, (3) TB+LTBI vs. HC+NMP, and (4) TB+NMP vs. HC+LTBI, were also determined (Table 4). The ROC analyses of IFN-γ measured in *M*.*tb*-stimulated whole blood QFT TB1 and QFT TB2 cultures showed the highest AUCs in the TB+LTBI v. NMP+HC comparison, 0.962 and 0.981 in TB1 QFT and TB2 QFT cultures, respectively (Table 4). In TB vs LTBI+NMP+HC comparison, the AUCs in QFT TB1 and QFT TB2 cultures were 0.724 and 0.729, respectively. AUC values for IFN-γ measured in sera were similar in each of the comparisons tested (Table 4).

### Gradient boosting based multiclass classification

To evaluate the potential to discriminate between study groups by all markers and select the most informative set of molecular and environmental factors we utilize a gradient boosting machine. We randomly divide the dataset into training and testing data (with ratio 7:3) and fit a gradient boosting based multiclass classifier with softmax objective function. We further evaluate the model based on 5-fold cross-validation procedure on training data only. In what follows, we fit the model on the entire training data and test predictions on the testing dataset. Using cross-validation, we estimate the accuracy to be 0.79 (95% CI = (0.7103, 0.8594) with highest balanced accuracy is achieved for the LTBI classification (0.92), and for HC (0.86). Whereas the lowest balanced accuracy is observed for NMP (0.61) and TB (0.55). We present the ensemble of measures of discriminatory potential in Table 5.

**Table 5:** Summary measures of classification based on 5-fold cross validation on the training data with gradient boosting machine.

Statistics	Group			
	HC	LTBI	NMP	TB
Sensitivity	0.9155	0.9355	0.2667	0.1250
Specificity	0.7963	0.9149	0.9545	0.9829
Positive Predictive Value	0.8553	0.7838	0.4444	0.3333
Negative Predictive Value	0.8776	0.9773	0.9052	0.9426
Precision	0.8553	0.7838	0.4444	0.3333
Recall	0.9155	0.9355	0.2667	0.1250
F1	0.8844	0.8529	0.3333	0.1818
Prevalence	0.5680	0.2480	0.1200	0.0640
Detection Rate	0.5200	0.2320	0.0320	0.0080
Detection Prevalence	0.6080	0.2960	0.0720	0.0240
Balanced Accuracy	0.8559	0.9252	0.6106	0.5540

Abbreviations: HC–*M*.*tb*-uninfected healthy controls; LTBI–latently *M*.*tb* infected individuals; NMP–patients with nonmycobacterial pneumonia; TB–active tuberculosis patients.

In what follows, we test the accuracy of the model on testing dataset. Our model achieves 0.87 accuracy (95% CI = (0.7421, 0.9441). Similarly as for the training data, we evaluate the per-class performance of the model, as measured by the balanced accuracy, to be highest for the HC class (0.96) and for the LTBI class (0.90), whereas lowest for the NMP (0.82) and TB (0.59). We present the measures of goodness of classification in Table 6.

**Table 6:** Summary measures of classification for the testing dataset with gradient boosting machine.

Statistics	Group			
	HC	LTBI	NMP	TB
Sensitivity	0.9677	0.8571	0.6666	0.2500
Specificity	0.9524	0.9474	0.9795	0.9375
Positive Predictive Value	0.9677	0.8571	0.6666	0.2500
Negative Predictive Value	0.9524	0.9474	0.9795	0.9375
Precision	0.9677	0.8571	0.6666	0.2500
Recall	0.9677	0.8571	0.6666	0.2500
F1	0.9677	0.8571	0.6666	0.2500
Prevalence	0.5962	0.2692	0.0576	0.0769
Detection Rate	0.5769	0.2308	0.0384	0.0192
Detection Prevalence	0.5962	0.2692	0.0576	0.0769
Balanced Accuracy	0.9601	0.9023	0.8231	0.5937

Abbreviations: HC–*M*.*tb*-uninfected healthy controls; LTBI–latently *M*.*tb* infected individuals; NMP–patients with nonmycobacterial pneumonia; TB–active tuberculosis patients.

We further evaluate the basic measures of informativeness of each feature in the models by three metrics: (1) gain, which represents the gain in the classification accuracy for splits associated with a particular feature, (2) cover, which is the percentage of the number of observations in leaves associated with particular feature, and (3) frequency, which is the percentage of the number of trees that the feature has been used in. These measures are presented in Table 7. We note that the three most informative features are: IFN-γ TB2 QFT, age and IFN-γ TB1 QFT.

**Table 7:** Summary of feature importance scores in the gradient boosting model.

Feature	Gain	Cover	Frequency
IFN-γ TB2 QFT	0.4902	0.2523	0.1793
age	0.1175	0.1611	0.1270
IFN-γ TB1 QFT	0.1155	0.1106	0.1076
IP-10 serum	0.0440	0.0709	0.0941
IP-10 TB1 QFT	0.0285	0.0606	0.0807
IP-10 urine	0.0218	0.0404	0.0553
IFN-γ serum	0.0211	0.0547	0.0582
IP-10 TB2 QFT	0.0185	0.0327	0.0224
sex	0.0085	0.0147	0.0313

Abbreviations: IFN-γ–interferon-gamma, IP-10 –IFN-γ inducible protein 10; QFT—QuantiFERON-TB Gold Plus assay.

We further compare the informativeness of IFN-γ vs IP-10-related predictors by building gradient boosting models separately on IFN-γ and IP-10. In Tables 8 and 9, we present summary measures for this comparison. We note that the model based on the IFN-γ achieves better accuracy for distinguishing the HC from the remaining groups and individuals in either TB or LTBI versus the two remaining classes. At the same time, we note that the mildly better performance of the model which discriminates between TB+ LTBI vs HC+NMLD does not translate to the better performance in distinguishing between TB and all other groups–both in terms of accuracy as well as sensitivity and specificity.

**Table 8:** Comparison of accuracy for binomial (logistic models) based on IFN-γ (top row) and IP-10 (bottom row). The balanced accuracy for each model for the training data is presented, together with the balanced accuracy for the testing data (in brackets).

Protein	HC vs. TB+LTBI+NMP	TB vs. LTBI+NMP+HC	TB+LTBI vs. HC+NMP	TB+NMP vs. HC+LTBI
IFN-γ	0.85 (0.96)	0.61 (0.73)	0.95 (0.96)	0.67 (0.81)
IP-10	0.76 (0.88)	0.61 (0.5)	0.87 (0.94)	0.65 (0.87)

Abbreviations: IFN-γ–interferon-gamma, IP-10 –IFN-γ inducible protein 10; HC–*M*.*tb*-uninfected healthy controls; LTBI–latently *M*.*tb* infected individuals; NMP–patients with nonmycobacterial pneumonia; TB–active tuberculosis patients.

**Table 9:** Comparison of sensitivity and specificity for binomial (logistic models) based on IFN-γ (top row) and IP-10 (bottom row). The results are presented as sensitivity/specificity for each model for the training data as well as for the testing data (in brackets).

Protein	HC vs. TB+LTBI+NMP	TB vs. LTBI+NMP+HC	TB+LTBI vs. HC+NMP	TB+NMP vs. HC+LTBI
IFN-γ	0.83/0.86 (0.95/0.97)	0.97/0.25 (0.95/0.5)	0.98/0.92 (0.97/0.94)	0.94/0.39 (0.91/0.71)
IP-10	0.67/0.86 (1/0.77)	0.97/0.25 (1/0.5)	0.92/0.82 (0.94/0.94)	0.91/0.39 (0.88/0.85)

Abbreviations: IFN-γ–interferon-gamma, IP-10 –IFN-γ inducible protein 10; HC–*M*.*tb-*uninfected healthy controls; LTBI–latently *M*.*tb* infected individuals; NMP–patients with nonmycobacterial pneumonia; TB–active tuberculosis patients.

## Discussion

This study suggests that IP-10 could be a viable alternative to IFN-γ as a biomarker for detecting *M*.*tb* infection in children vaccinated with BCG, especially in regions where TB is not common. However, it should be noted that IP-10 does not distinguish between active and latent *M*.*tb* infection.

IP-10, derived from monocytes/macrophages, directs Th1 lymphocytes to inflammation sites via the CXCR3 receptor and is induced by IFN-γ, linking it to the immune response to M.tb infection [20–23]. Its elevated levels in TB patients’ pleural effusions and pulmonary granulomas suggest diagnostic potential [24–26]. IP-10’s sensitivity as a marker, particularly after M.tb antigen stimulation, is well documented and unaffected by HIV-related factors like CD4 count or mitogen reactivity [27]. However, its ability to distinguish between active TB and LTBI remains debated, despite some studies supporting its diagnostic utility in differentiating between the two [28, 29]. Our results show elevated IP-10 levels in both blood cultures stimulated with M.tb antigens and urine of children with active or latent TB, compared to uninfected individuals. This supports IP-10’s potential in identifying *M*.*tb* infections in children, aligning with previous findings. We observed consistent IP-10 increases across different QFT blood cultures, consistent with Petrone et al [15]. In contrast, no differences were found in unstimulated blood, unlike Fisher et al., who reported higher IP-10 levels in M.tb-infected individuals [20]. Discrepancies may stem from study design, participant differences, and detection methods, highlighting the need for standardized approaches in future research.

To our knowledge, this study is the first to report a significant increase in urinary IP-10 levels in children with *M*.*tb* infection in TB low-prevalence areas. Previous research mostly focused on adults or found no link between urinary IP-10 and *M*.*tb* infection [12, 15, 30, 31]. Petrone et al. found IP-10 detectable in children with TB but concluded it doesn’t effectively distinguish TB from other respiratory diseases, suggesting IP-10 may be better suited as an inflammatory marker [15]. Other studies, like those by Kim and Sukumar, indicate that urinary IP-10 could be useful in tracking treatment efficacy [30]. The ability to detect IP-10 in urine, irrespective of age, highlights its potential as a marker of inflammation, particularly as it offers a more accessible means of assessment compared to other conventional diagnostic indicators. Urinary IP-10 offers a promising, non-invasive approach for diagnosing TB; however, further studies with larger sample sizes are needed to fully evaluate its sensitivity and specificity in differentiating TB infection statuses.

The concordance between IP-10 and IFN-γ has been widely studied for improving TB detection, particularly in distinguishing active TB from latent infection. In our study, IFN-γ and IP-10 levels were strongly correlated, and both are recognized as valuable TB biomarkers. Studies, like Blauenfeldt et al., demonstrated high specificity and sensitivity of IP-10 assays, though QFT slightly outperformed them [32]. A meta-analysis to evaluate the diagnostic accuracy of IP-10 for distinguishing active TB from latent TB infection, adjusting various factors, including country economic status, assay condition (M.tb antigen-stimulated/unstimulated), study design, HIV status, and IP-10 cut-off levels showed the significant discriminatory effect of IP-10, suggesting that IP-10 is a good biomarker for TB diagnosis [33]. It is suggested that the synergy between IFN-γ and IP-10 potentially enriches the diagnostic toolkit for TB, paving the way for more refined assays capable of identifying both active TB and latent TB infection with greater sensitivity and specificity. Several studies have explored the relationship between IFN-γ, IP-10, and the tuberculin skin test (TST) in detecting *M*.*tb* infection in children. Research in Ethiopia found that children exposed to adults with high *M*.*tb* bacilli levels were more likely to test positive for TST, IFN-γ, and IP-10 than healthy controls [34]. Petrucci et al. reported strong concordance between TST and QFT, with higher IP-10 responses in children positive for both [35]. Notably, children with negative TST but positive QFT had elevated IP-10 levels, suggesting its potential for identifying latent TB. These findings support a combined testing approach using IP-10 to improve TB diagnosis, particularly in children.

Our study had several limitations. The small sample size raises concerns about statistical power and type II errors, though the detailed participant characterization offers valuable insights for future research. The lack of a gold standard for diagnosing TB in children may have introduced population bias, and while strict criteria aimed to reduce misclassification, it cannot be entirely excluded. Additionally, the high variability of results indicating underlying biological differences, potential confounders, or varying degrees of immune system activation complicates the interpretation of findings, emphasizing the need for larger, more homogeneous populations and improved diagnostic tools to better understand immune responses in TB.

Our study highlights several key points for future research. First, the study identifies IP-10 as a specific biomarker for TB infection, suggesting its potential utility in TB diagnosis. However, it also notes that IP-10 does not outperform IFN-γ in distinguishing between active TB disease and latent TB infection, suggesting that it should not be considered as a stand-alone diagnostic tool. Secondly, our results suggest that while IP-10 alone does not provide a definitive distinction between active TB and LTBI, its diagnostic performance may be improved when used in combination with other markers. This suggests that future diagnostic strategies may benefit from incorporating IP-10 alongside other biomarkers to improve accuracy. Third, the study highlights the need for further research using larger populations and more standardised assay techniques for IP-10. This would help to confirm the findings and potentially refine the use of IP-10 as a diagnostic marker for TB.

## Conclusions

IP-10 shows promise as a biomarker for *M*.*tb* infection in BCG-vaccinated children, though it has limitations in distinguishing active TB from LTBI. Further validation in larger studies and optimization of its diagnostic use, including combining it with other biomarkers and standardizing techniques, is needed for TB management.

## Supporting information

S1 Fig Lack of correlation between the age of the children studied and the levels of IP-10 or IFN-γ measured in serum (S1A, S1B), urine (S1C), QFT TB1 (S1D, S1E) and QFT TB2 (S1F, S1G) cultures. (A) Spearman rank correlation (rs) of age of children and serum IP-10 levels (r = 0.01, p = 0.83), (B) Spearman rank correlation (rs) of age of children and serum IFN-γ levels (rs = 0.07, p = 0.27), (C) Spearman rank correlation (rs) of age of children and urine IP-10 levels (rs = -0.05, p = 0.4), (D) Spearman rank correlation (rs) of age of children and QFT TB1 IP-10 levels (rs = 0.1, p = 0.14), (E) Spearman rank correlation (rs) of age of children and QFT TB1 IFN-γ levels (rs = 0.05, p = 0.39), (F) Spearman rank correlation (rs) of age of children and QFT TB2 IP-10 levels (rs = 0.08, p = 0.20), (G) Spearman rank correlation (rs) of age of children and QFT TB2 IFN-γ levels (rs = 0.08, p = 0.19). Abbreviations: IFN-γ–interferon-gamma, IP-10 –IFN-γ inducible protein 10. Statistical analysis was performed using the Spearman’ rank correlation test and p value was considered significant if < 0.05.
(TIF)

S2 Fig The violin plots for the tuberculin skin test (TST) size in the studied groups. The shape of the plot represents the fitted density of the TST distribution in the respective group. Differences between the groups were compared using the non-parametric one-tailed ANOVA. A p value was considered significant if < 0.05. Abbreviations: HC–healthy controls, IP-10 –IFN-γ inducible protein 10, LTBI–latent *M*.*tb* infection, NMP–nonmycobacterial lung disease, TB- tuberculosis.
(TIF)

S3 Fig ROC curves for the IP-10 or IFN-γ measured in serum, urine and QFT cultures for the differentiation of TB patients from other groups (LTBI+NMP+HC). A) ROC curve for the serum IP-10 levels, (B) ROC curve for the QFT TB1 IP-10 levels, (C) ROC curve for the QFT TB2 IP-10 levels, (D) ROC curve for the urine IP-10 levels, (E) ROC curve for the serum IFN-γ levels, (F) ROC curve for the QFT TB1 IFN-γ levels, (G) ROC curve for the QFT TB2 IFN-γ levels.
(TIF)

S4 Fig ROC curves for the IP-10 or IFN-γ measured in serum, urine and QFT cultures for the differentiation of *M*.*tb*-infected individuals (TB+LTBI) from other groups (HC+NMP). A) ROC curve for the serum IP-10 levels, (B) ROC curve for the QFT TB1 IP-10 levels, (C) ROC curve for the QFT TB2 IP-10 levels, (D) ROC curve for the urine IP-10 levels, (E) ROC curve for the serum IFN-γ levels, (F) ROC curve for the QFT TB1 IFN-γ levels, (G) ROC curve for the QFT TB2 IFN-γ levels.
(TIF)

S5 Fig ROC curves for the IP-10 or IFN-γ measured in serum, urine and QFT cultures for the differentiation of patients with pneumonia (TB+NMP) from other groups (HC+LTBI). A) ROC curve for the serum IP-10 levels, (B) ROC curve for the QFT TB1 IP-10 levels, (C) ROC curve for the QFT TB2 IP-10 levels, (D) ROC curve for the urine IP-10 levels, (E) ROC curve for the serum IFN-γ levels, (F) ROC curve for the QFT TB1 IFN-γ levels, (G) ROC curve for the QFT TB2 IFN-γ levels.
(TIF)

S6 Fig ROC curves for the IP-10 or IFN-γ measured in serum, urine and QFT cultures for the differentiation of healthy controls (HC) from other groups (TB+LTBI+NMP). A) ROC curve for the serum IP-10 levels, (B) ROC curve for the QFT TB1 IP-10 levels, (C) ROC curve for the QFT TB2 IP-10 levels, (D) ROC curve for the urine IP-10 levels, (E) ROC curve for the serum IFN-γ levels, (F) ROC curve for the QFT TB1 IFN-γ levels, (G) ROC curve for the QFT TB2 IFN-γ levels.
(TIF)
