# Supplementary figures and images for: Interferon (IFN)-gamma (γ) inducible protein 10 (IP-10) in the diagnosis of latent and active tuberculosis in Bacille Calmette Guerin (BCG)-vaccinated pediatric population
Source: PLoS One. 2025 Jan 21;20(1):e0314400. doi: 10.1371/journal.pone.0314400 (PMC11750100; doi:10.1371/journal.pone.0314400)

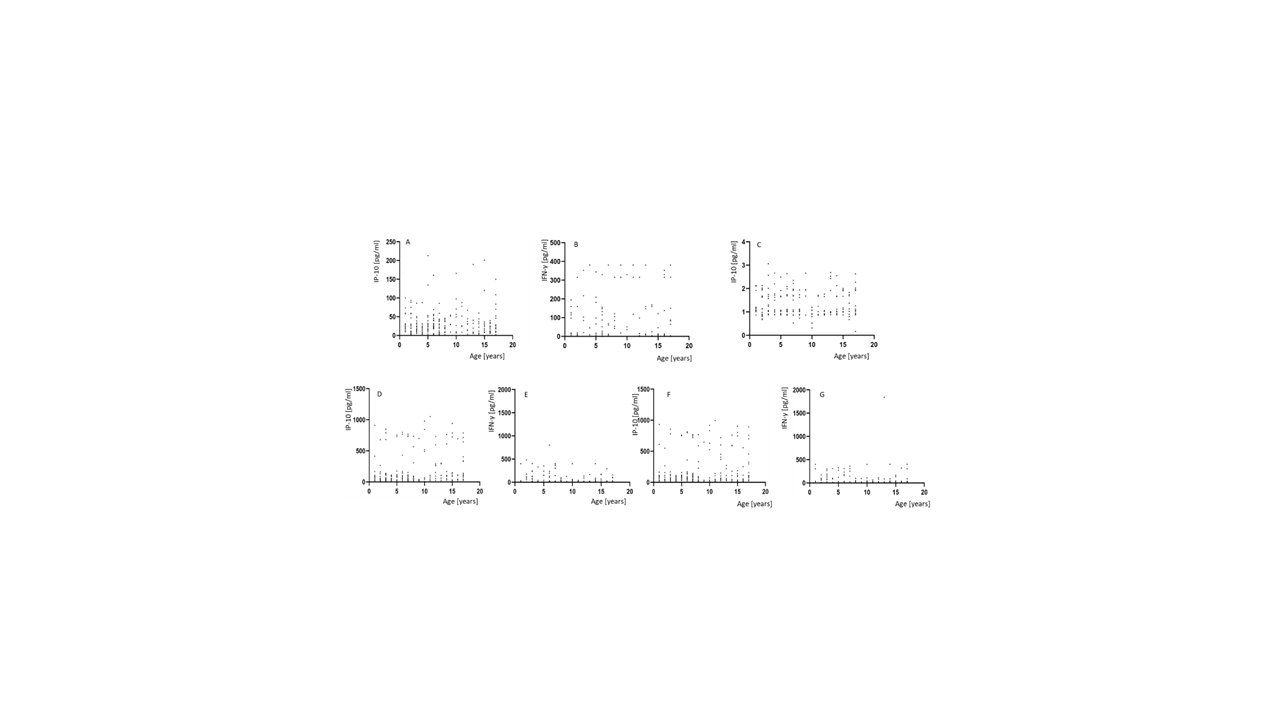

Supplement: S1 Fig — (A) Spearman rank correlation (rs) of age of children and serum IP-10 levels (r = 0.01, p = 0.83), (B) Spearman rank correlation (rs) of age of children and serum IFN-γ levels (rs = 0.07, p = 0.27), (C) Spearman rank correlation (rs) of age of children and urine IP-10 levels (rs = -0.05, p = 0.4), (D) Spearman rank correlation (rs) of age of children and QFT TB1 IP-10 levels (rs = 0.1, p = 0.14), (E) Spearman rank correlation (rs) of age of children and QFT TB1 IFN-γ levels (rs = 0.05, p = 0.39), (F) Spearman rank correlation (rs) of age of children and QFT TB2 IP-10 levels (rs = 0.08, p = 0.20), (G) Spearman rank correlation (rs) of age of children and QFT TB2 IFN-γ levels (rs = 0.08, p = 0.19). Abbreviations: IFN-γ–interferon-gamma, IP-10 –IFN-γ inducible protein 10. Statistical analysis was performed using the Spearman’ rank correlation test and p value was considered significant if < 0.05. (TIF) [file pone.0314400.s001.tif]

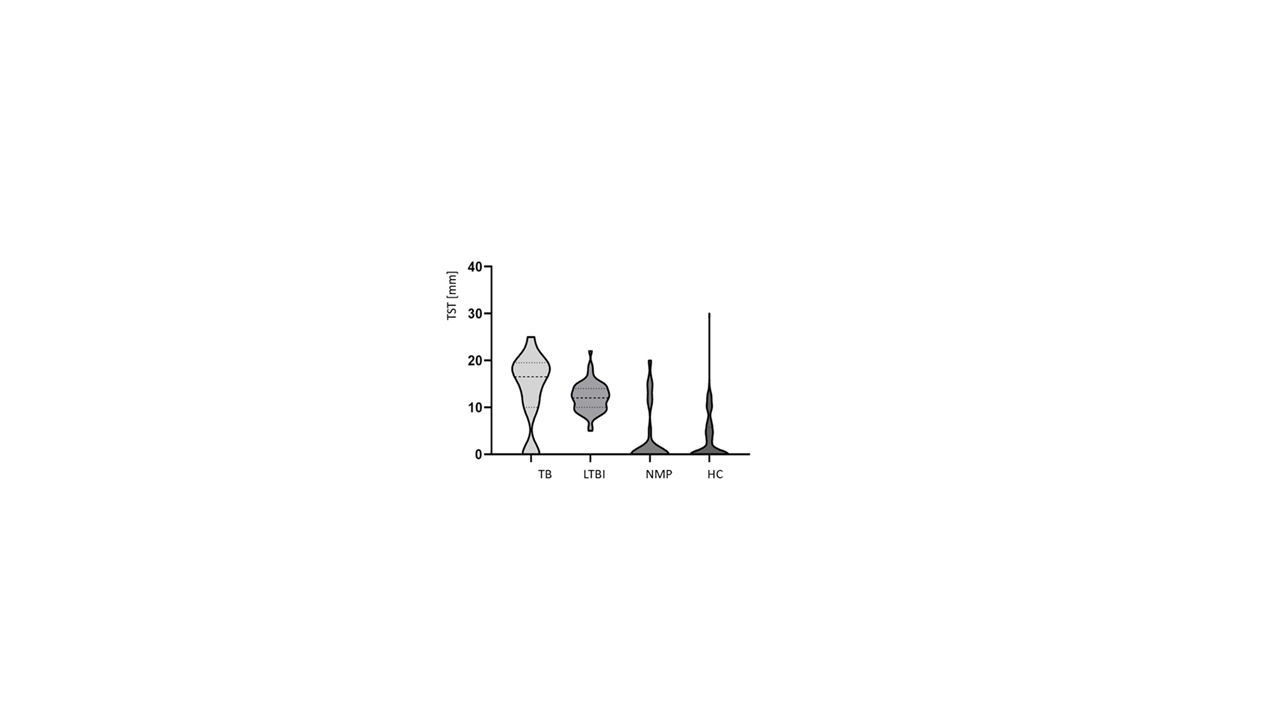

Supplement: S2 Fig — The shape of the plot represents the fitted density of the TST distribution in the respective group. Differences between the groups were compared using the non-parametric one-tailed ANOVA. A p value was considered significant if < 0.05. Abbreviations: HC–healthy controls, IP-10 –IFN-γ inducible protein 10, LTBI–latent M.tb infection, NMP–nonmycobacterial lung disease, TB- tuberculosis. (TIF) [file pone.0314400.s002.tif]

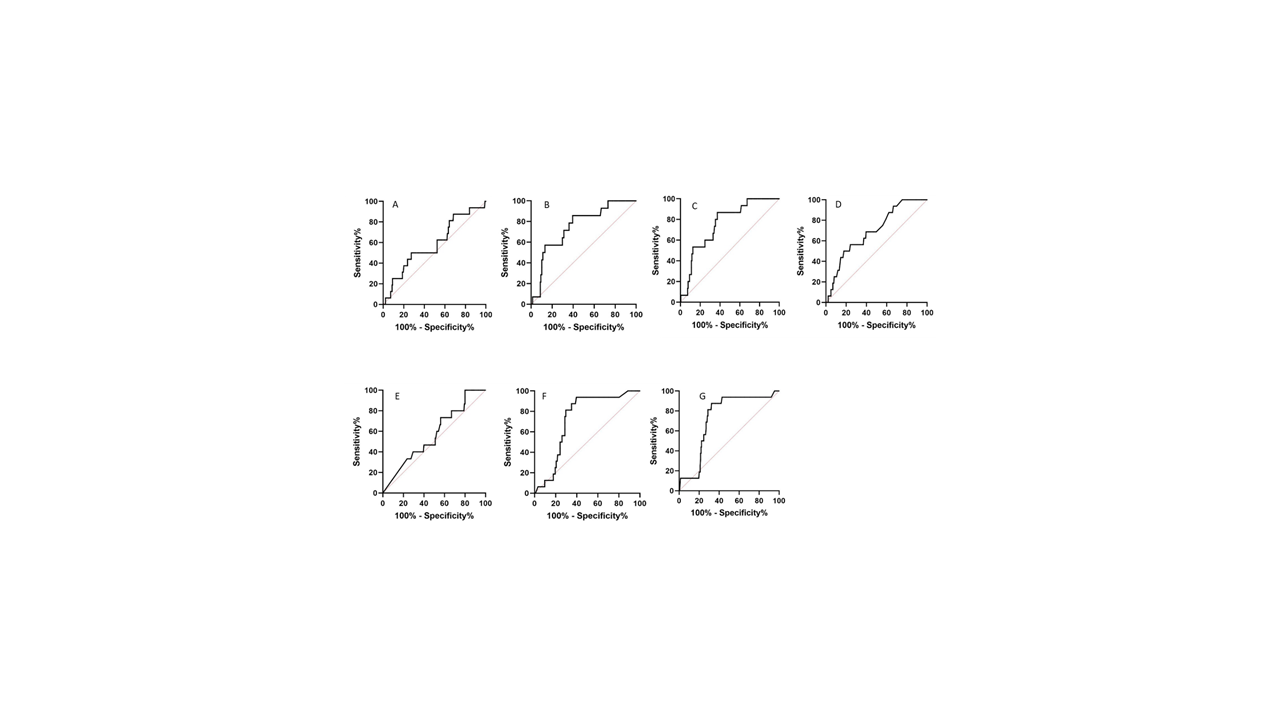

Supplement: S3 Fig — A) ROC curve for the serum IP-10 levels, (B) ROC curve for the QFT TB1 IP-10 levels, (C) ROC curve for the QFT TB2 IP-10 levels, (D) ROC curve for the urine IP-10 levels, (E) ROC curve for the serum IFN-γ levels, (F) ROC curve for the QFT TB1 IFN-γ levels, (G) ROC curve for the QFT TB2 IFN-γ levels. (TIF) [file pone.0314400.s003.tif]

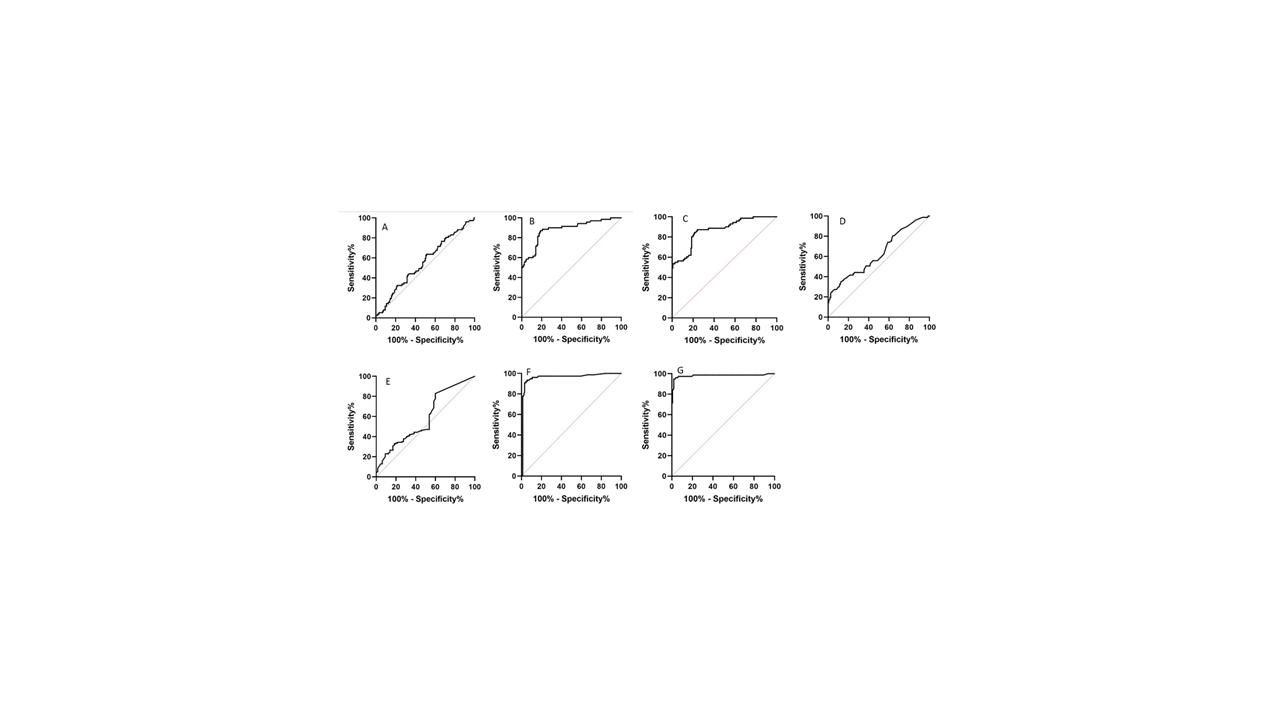

Supplement: S4 Fig — A) ROC curve for the serum IP-10 levels, (B) ROC curve for the QFT TB1 IP-10 levels, (C) ROC curve for the QFT TB2 IP-10 levels, (D) ROC curve for the urine IP-10 levels, (E) ROC curve for the serum IFN-γ levels, (F) ROC curve for the QFT TB1 IFN-γ levels, (G) ROC curve for the QFT TB2 IFN-γ levels. (TIF) [file pone.0314400.s004.tif]

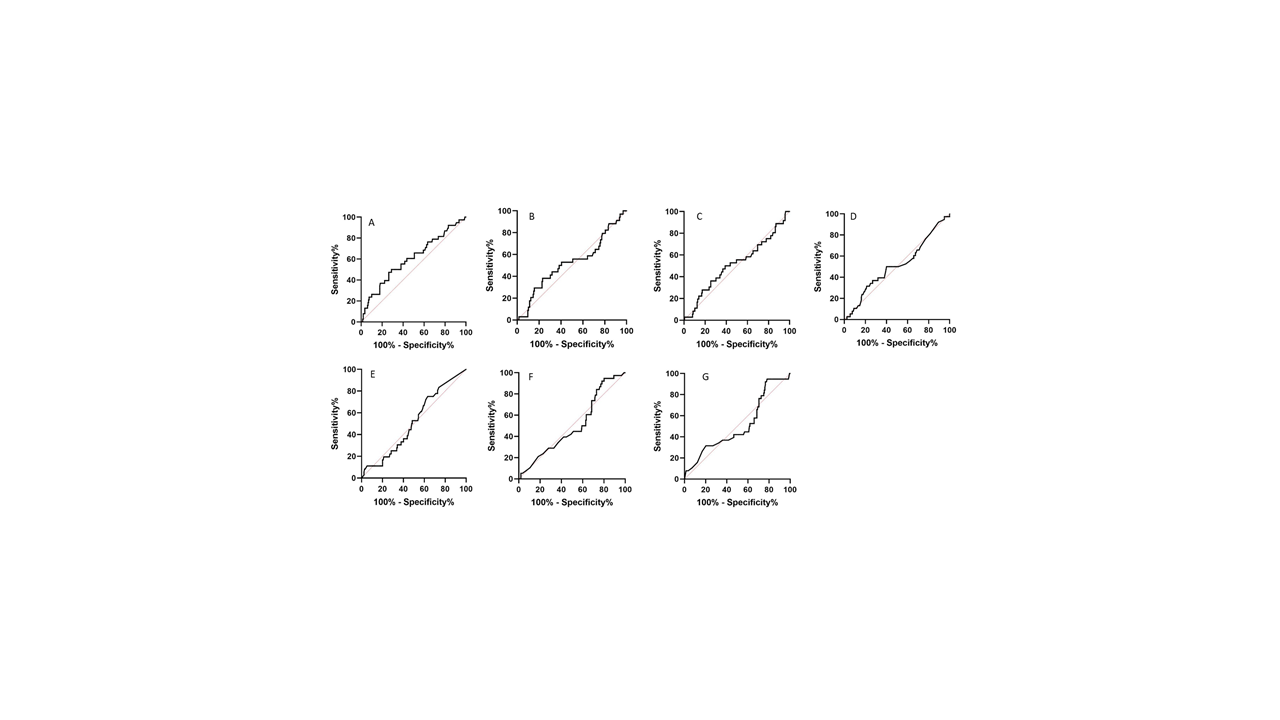

Supplement: S5 Fig — A) ROC curve for the serum IP-10 levels, (B) ROC curve for the QFT TB1 IP-10 levels, (C) ROC curve for the QFT TB2 IP-10 levels, (D) ROC curve for the urine IP-10 levels, (E) ROC curve for the serum IFN-γ levels, (F) ROC curve for the QFT TB1 IFN-γ levels, (G) ROC curve for the QFT TB2 IFN-γ levels. (TIF) [file pone.0314400.s005.tif]

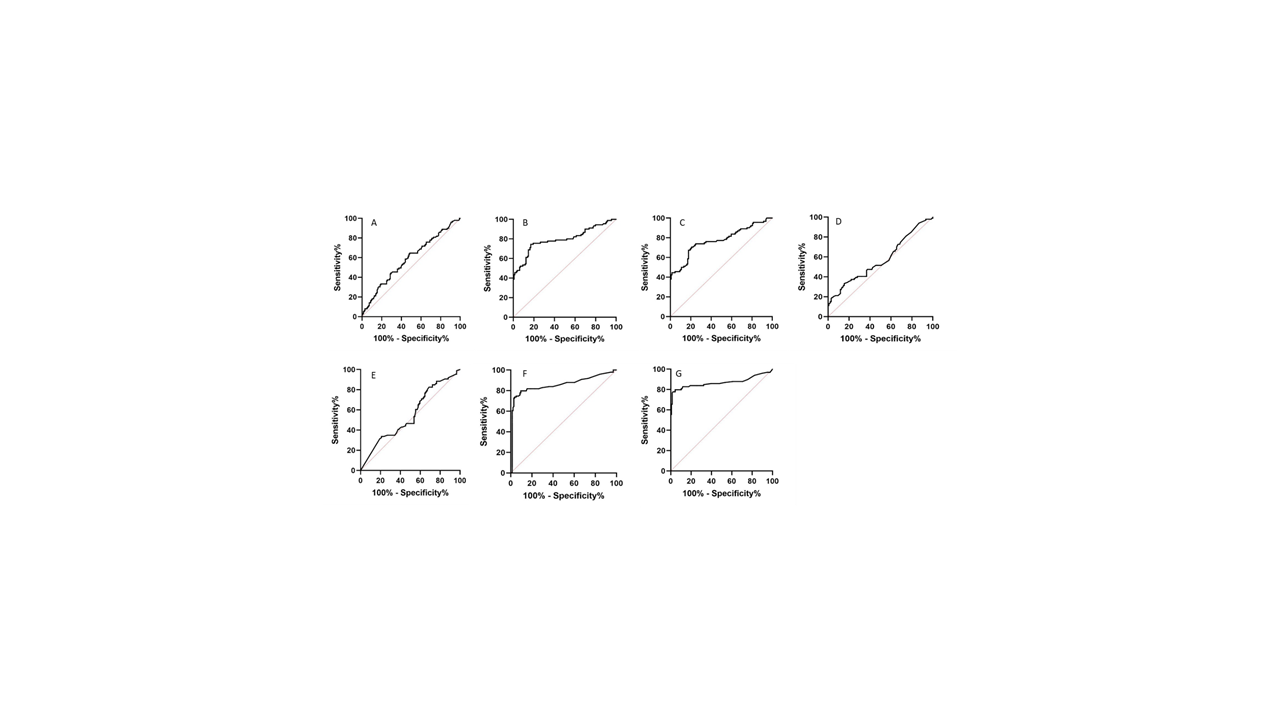

Supplement: S6 Fig — A) ROC curve for the serum IP-10 levels, (B) ROC curve for the QFT TB1 IP-10 levels, (C) ROC curve for the QFT TB2 IP-10 levels, (D) ROC curve for the urine IP-10 levels, (E) ROC curve for the serum IFN-γ levels, (F) ROC curve for the QFT TB1 IFN-γ levels, (G) ROC curve for the QFT TB2 IFN-γ levels. (TIF) [file pone.0314400.s006.tif]
